# The investigation of the basic psychological needs of athletes in the Turkish National Amputee Football Team: a qualitative study

**DOI:** 10.3389/fpsyg.2026.1855925

**Published:** 2026-07-09

**Authors:** Ersan Kara

**Affiliations:** Department of Coaching Education, Faculty of Sport Sciences, Kırşehir Ahi Evran University, Kırşehir, Türkiye

**Keywords:** adapted physical activity, amputee football, basic psychological needs, disabled athletes, psychological well-being, self-determination theory, social belonging

## Abstract

**Introduction:**

The aim of this study is to examine the experiences of athletes in the Turkish Amputee National Football Team regarding the dimensions of autonomy, competence, and relatedness, which are their basic psychological needs.

**Method:**

The research was designed within the framework of a qualitative research approach, and the participant group consists of nine athletes who are actively involved in the Turkish Amputee National Football Team. The participants consist of individuals with different levels of amputation. The data were collected through semi-structured interviews and observations conducted during training and competition processes. The interviews were conducted face-to-face, audio-recorded, and later transcribed into written texts. The obtained data were analyzed using the thematic analysis method. As a result of the analysis, five themes emerged: (1) autonomy and freedom of decision-making, (2) perception of competence and sense of achievement, (3) social bonds and belonging, (4) coping with disability and psychological resilience, and (5) performance pressure and internal-external tension.

**Results:**

The findings show that athletes’ motivation and self-confidence increase when their autonomy is supported; however, external pressures can limit this process. It was determined that the perception of competence is strengthened through performance opportunities and experiences of success, while social bonds are shaped through belonging, trust, and feeling valued. However, it was also observed that belonging is not always inclusive and is constructed through selective relationships. In addition, the findings reveal that psychological resilience is not only an individual trait but is shaped by social relationships and the sporting context. It was determined that athletes continuously try to maintain a balance between performance expectations and their intrinsic motivation, and that this situation sometimes creates tension.

**Discussion:**

The findings further demonstrate that amputee football simultaneously functions as a context of empowerment and psychological pressure, where athletes negotiate autonomy, competence, and belonging under conditions shaped by performance expectations and social evaluation. As a result, the study reveals that amputee football is both an empowering and limiting field of experience for athletes, and that sports environments should be restructured in a way that supports psychological needs.

## Introduction

According to data from the World Health Organization (WHO), approximately 16% of the world’s population, that is, more than 1.3 billion people, live with some form of disability (World Health Organization [WHO], 2023). In the context of Türkiye, the proportion of individuals with disabilities is reported as 12.3%, and a significant portion of this group consists of individuals with physical disabilities (Turkish Statistical Institute [TURKSTAT], 2002). On a global scale, it is reported that the number of individuals with amputations is approximately 40 million, and the vast majority of these amputations involve the lower extremities (World Health Organization [WHO], 2017). In Türkiye, it is estimated that there are approximately 300,000 individuals with amputations (Topuz et al., 2011). These data indicate that individuals with physical disabilities, and especially those who have experienced amputation, represent a significant population both globally and nationally. However, it is observed that the level of participation in physical activity among individuals with disabilities is significantly lower compared to the general population (World Health Organization [WHO], 2021). Many studies have revealed that individuals with physical disabilities have more sedentary lifestyles and that their participation in sports activities remains limited (Sansi and Çetin, 2025; Barbosa-Granados et al., 2024; Jang et al., 2023; Isidoro-Cabanas et al., 2023; Bentzen et al., 2021; Martin Ginis et al., 2021; Sæbu et al., 2013; Rimmer and Marques, 2012; Beyazoğlu and Özbek, 2025). In addition, it is emphasized that individuals with disabilities cannot reach the recommended levels of physical activity and that a sedentary lifestyle is prevalent in this group (World Health Organization [WHO], 2022; Rimmer et al., 2004). This situation leads to significant negative consequences not only in terms of physical health but also in terms of psychological well-being and social adaptation (Darabi et al., 2025; Zabala-Domínguez et al., 2024).

Participation in physical activity and sports is supported by a wide body of literature as having positive effects on both the physical and psychological health of individuals (Warburton et al., 2006; Eime et al., 2013; Liu and Zhong, 2023; Zhang et al., 2023). These effects are not limited to physiological gains; they also include important psychological outcomes such as increased self-esteem (Fox, 1999; Martin Ginis et al., 2016; Li et al., 2025), development of positive affect (Cid et al., 2025; Toros et al., 2023; Beyazoğlu and Haegele, 2025), and enhanced life satisfaction (Warburton et al., 2006; Ağduman and Daşkesen, 2023). Especially for individuals with disabilities, sport is considered not only a physical activity but also an important component of identity reconstruction, social acceptance, and psychological empowerment processes (Rasheedi and Rahimi, 2025; Abdelkader et al., 2022; Bentzen and Malmquist, 2022). Previous studies have also emphasized that participation in disability sport may contribute to psychological well-being through mechanisms such as social connectedness, competence development, and increased self-efficacy (Jaarsma et al., 2014; Banack et al., 2011; Martin Ginis et al., 2016).

However, when the literature in this field is examined, some important limitations stand out. First, studies explaining physical activity motivation in individuals with disabilities are limited (Sæbu et al., 2013). Additionally, it is stated that studies addressing the psychological processes of athletes with disabilities in the sports psychology literature are both few in number and lack methodological depth (Barbosa-Granados et al., 2024). Furthermore, it is observed that a large portion of existing studies either focus on non-disabled athletes or address heterogeneous disability groups together (Hebeş and İnce, 2025; Darisman et al., 2025; Wieczorek et al., 2017). This situation indicates that the unique experiences of amputee athletes, in particular, have not been examined in sufficient depth. Rather than simply confirming positive motivational outcomes, there remains a need for qualitative research examining how elite disabled athletes simultaneously experience empowerment, pressure, vulnerability, and selective belonging within competitive sport environments.

Moreover, it is noteworthy that intervention and theoretical frameworks supporting psychological well-being have not been addressed in a sufficiently holistic manner in the existing literature (Zabala-Dominguez et al., 2023). At this point, Self-Determination Theory (SDT) provides an important theoretical framework. According to this theory, three basic psychological needs–autonomy, competence, and relatedness–must be satisfied in order to sustain individuals’ psychological well-being (Ryan and Deci, 2017). Recent developments in SDT further emphasize that the absence of need satisfaction is conceptually different from the active frustration or thwarting of psychological needs (Bartholomew et al., 2011; Vansteenkiste et al., 2020). In sport contexts, athletes may simultaneously experience moments of need satisfaction and need frustration, particularly under controlling interpersonal conditions, performance pressure, and selective social relationships. In this regard, SDT has increasingly shifted from a purely motivational framework toward a perspective that also examines tension, contradiction, and contextual vulnerability in athletes’ experiences.

Sports environments offer important opportunities for the satisfaction of these needs and can enhance individuals’ motivation, performance, and well-being levels (Teixeira et al., 2012). However, how these needs are experienced and under what conditions they are supported among athletes with disabilities has not yet been sufficiently clarified. In particular, how basic psychological needs are satisfied among individuals with physical disabilities who compete at an elite level and how these processes are related to the sport experience have been addressed only to a limited extent in the literature (Ağduman and Daşkesen, 2023; Van Yperen, 2025). Existing findings suggest that as sport experience increases, perceptions of autonomy may strengthen, and that the level of competition may influence experiences of competence and relatedness (Teixeira et al., 2012; Zhang and Miao, 2024). However, how these relationships are shaped specifically within the context of competitive and team-based sports such as amputee football is not yet fully understood.

It is known that individuals who have undergone amputation experience significant changes in physical functionality, psychological well-being, and social life (Bragaru et al., 2011). In particular, lower extremity amputation is associated with outcomes such as loss of mobility, changes in body image, and reduced social participation (Güçhan Topçu et al., 2017). This process makes psychological adjustment more difficult and may lead to increased levels of depression and anxiety (Horgan and MacLachlan, 2004). In this context, sport emerges as an important component not only of physical rehabilitation but also of psychological and social recovery for individuals who have experienced amputation (Webster et al., 2001; Bragaru et al., 2011). Participation in adapted sports has been shown to have positive effects on quality of life, self-esteem, and life satisfaction (Yazıcıoğlu et al., 2012; Auricchio et al., 2017; Jaarsma et al., 2014).

Among adapted sports, amputee football draws attention due to its dynamic structure and team-based characteristics (Monteiro, 2014). This sport not only provides individuals with opportunities for physical activity but also offers important opportunities in terms of social interaction, sense of belonging, and identity development (Telepbergen and Ermiş, 2024). However, the experiences of amputee football players are not limited to positive aspects. These athletes also face various psychological challenges such as social stigma, performance pressure, and fear of re-injury (McDonald, 2021; Hebeş and İnce, 2025). This situation indicates that amputee football players constitute a group that should be specifically examined within the context of sports psychology.

When all this literature is evaluated together, although there are important findings regarding the positive effects of sport on individuals with disabilities, it is seen that studies systematically examining the basic psychological needs of amputee football players within the framework of Self-Determination Theory are limited. However, there is a lack of in-depth qualitative research examining how elite-level amputee football players experience autonomy, competence, and relatedness needs and how these needs are related to sport participation. More importantly, existing studies have generally approached these needs through linear and confirmatory assumptions, focusing predominantly on positive outcomes such as motivation, well-being, and participation. As a result, less attention has been paid to the contradictory and context-dependent nature of psychological need experiences among disabled athletes, particularly in relation to need frustration, selective belonging, fragile competence, and autonomy constructed through resistance.

In this context, the present study aims to examine the basic psychological needs of amputee football players. Beyond identifying whether psychological needs are satisfied, this study aims to contribute to SDT literature by examining how these needs are simultaneously supported, restricted, negotiated, and reconstructed within elite amputee football contexts. In doing so, the study conceptualizes sport not only as a motivational environment but also as a socially and structurally shaped space in which psychological needs may coexist with vulnerability, tension, and pressure. This study aims to contribute to the mechanisms explaining the effects of sport participation on psychological well-being and to provide an important perspective for both the sports sciences literature and practitioners. In line with this, the sub-objectives of the study can be expressed as follows: (1) to examine the autonomy experiences of amputee football players, (2) to reveal how perceptions of competence develop in the sports environment, and (3) to analyze relatedness experiences within the context of team and social environment.

### Conceptual framework

The concept of basic psychological needs is addressed within the framework of Self-Determination Theory (SDT), developed by Deci and Ryan (2000), Ryan and Deci (2017). SDT provides a comprehensive theoretical structure that explains an individual’s motivational processes, psychological development, and well-being, and places three basic psychological needs at the center of these processes. These can be defined as autonomy, competence, and relatedness (Deci and Ryan, 2000; Ryan and Deci, 2017). These needs are inherent in human nature and are considered universal; when satisfied, they provide the psychological support necessary for healthy development and well-being (Vansteenkiste et al., 2020; Li et al., 2025).

The need for autonomy is related to the individual’s sense of being the agent of their own behavior and acting in accordance with their own values (Deci and Ryan, 2000). In the context of sports, autonomy is supported through the athlete’s participation in decision-making processes and having choices (Balaguer et al., 2012). In addition, the autonomy support provided by individuals within the social environment strengthens the individual’s capacity to regulate their own behavior, thereby supporting the development of more internalized and sustainable forms of motivation (Slemp et al., 2024).

The need for competence refers to the individual’s feeling of being effective in their interactions with the environment, overcoming challenges, and developing mastery (Ryan and Deci, 2017). In sports settings, performance feedback and experiences of progress stand out as key factors that strengthen the perception of competence (Shannon et al., 2023). However, the individual’s perception of competence is not only related to performance but also to a supportive social environment; in this context, family support and psychological need support can play a determining role in the individual’s academic and athletic achievements (Scislo, 2018). In this regard, for athletes with disabilities, the sense of competence is not only an indicator of performance but also a fundamental psychological resource that reconstructs the perception of “I can succeed” beyond physical limitations and shapes the individual’s sport participation, self-confidence, and identity development.

The need for relatedness refers to the individual’s need to establish meaningful connections with others, to feel accepted, and to experience a sense of belonging (Deci and Ryan, 2000). In team sports, this need is satisfied through trust-based relationships established with teammates and coaches (Balaguer et al., 2012). Strengthening social bonds is considered one of the key mechanisms that enhance an individual’s psychological well-being (Li et al., 2025). There is an important conceptual distinction between the satisfaction of basic psychological needs and need frustration. While need satisfaction is positively associated with well-being and self-determined motivation (Slemp et al., 2024), need frustration refers to the active suppression of these needs by the social environment (Bartholomew et al., 2011). In particular, controlling interpersonal styles can lead to negative outcomes such as burnout, anxiety, and poor psychological health among athletes (Bartholomew et al., 2011; Shannon et al., 2023). Therefore, low need satisfaction and active need frustration should be conceptually distinguished from each other (Vansteenkiste et al., 2020). This distinction is particularly important in elite sport settings, where athletes may simultaneously experience empowerment and psychological pressure. In this regard, autonomy may coexist with forms of resistance against control, competence may remain fragile under constant evaluation, and relatedness may become conditional or selective depending on social acceptance processes. Thus, psychological needs should not be understood as static or uniformly positive experiences, but as dynamic processes continuously shaped by contextual and relational conditions.

For individuals with physical disabilities, sport is not only a physical activity but also a critical component of the psychological reconstruction process. Especially among amputee athletes, the loss of self-identity experienced after injury can be reconstructed through sport (Jordan et al., 2024). The sports environment restores a sense of control by providing autonomy experiences, reinforces the belief of “I can succeed” by strengthening the sense of competence, and supports the sense of belonging through social relationships (Jordan et al., 2024). In this context, the interpersonal style of coaches plays a decisive role in the satisfaction of athletes’ basic psychological needs. Autonomy-supportive coaching behaviors increase need satisfaction through practices such as taking the athlete’s perspective into account, offering choices, and explaining the rationale behind behaviors (Slemp et al., 2024). In contrast, controlling coaching approaches lead to need frustration and weaken motivational processes (Bartholomew et al., 2011).

In conclusion, in analyzing the experiences of athletes in the Turkish Amputee National Football Team, examining basic psychological needs provides a critical theoretical framework not only for understanding individual motivational processes but also for understanding the rehabilitative, identity-building, and social integration functions of sport.

## Materials and methods

In this study, a qualitative method was preferred. Qualitative research is used to understand a phenomenon and to obtain in-depth information about it. This study focuses on the experiences of athletes in the Turkish Amputee National Football Team and aims to examine how the basic psychological needs of autonomy, competence, and relatedness are experienced in the context of sport and the effect of amputee football on these needs. This study aims to expand the literature on the basic psychological needs of amputee athletes and to fill the gap in the existing literature regarding how these needs are experienced in the context of sport.

### Participants

The participants of this study consist of amputee football athletes who were determined in line with the principle of data saturation (Morse, 2010) and criteria appropriate to the research purpose (Creswell, 2003), and who voluntarily participated in the study. Criterion sampling method was used in the selection of the sample. Accordingly, the inclusion criteria for participants were determined as: (a) actively participating in amputee football, (b) being a member of the Turkish Amputee National Football Team, and (c) voluntary participation in the study. The participant group of the study consists of a total of 9 athletes who are actively involved in the Turkish Amputee National Football Team. The participants have different levels of amputation (below-knee, through-knee, and above-knee amputation), which ensures that the sample has a heterogeneous structure (Table 1). The ages of the participants range between 18 and 25, and all participants are male athletes (Table 1). While the majority of the athletes have been participating in sports at a professional level for many years, some participants have shorter-term sports experience. In accordance with ethical principles, the identities of the participants were kept confidential during the research process, and each participant was coded differently and included in the analysis process.

**TABLE 1:** Participant demographic information and amputation characteristics.

Name (Pseudonym)	Age	Gender	Amputation type
Can	22	Male	Transtibial (below-knee amputation)
Mehmet	25	Male	Knee disarticulation
Ali	18	Male	Knee disarticulation
Musa	19	Male	Knee disarticulation
Eser	22	Male	Transtibial (below-knee amputation)
Özgür	22	Male	Transtibial (below-knee amputation)
Aslan	24	Male	Transtibial (below-knee amputation)
Yasin	18	Male	Transfemoral (above-knee amputation)
Erkan	23	Male	Transfemoral (above-knee amputation)

### Procedures

The ethical approval of the study was obtained from the Scientific Research and Publication Ethics Committee of Social and Human Sciences at Kırşehir Ahi Evran University. Within the scope of the study, athletes actively participating in the Turkish Amputee National Football Team were contacted and invited to participate in the study on a voluntary basis. Nine athletes who met the specified criteria agreed to participate in the study. Prior to the data collection process, the purpose, scope, and procedures of the research were explained in detail to the participants, and they were asked to read and approve the informed consent form.

### Data collection

During the data collection process, semi-structured interview questions were prepared based on the basic psychological needs within the framework of Self-Determination Theory and in line with the relevant literature. The interview form was developed to explore in depth the sport experiences, motivational processes, and social interactions of amputee football athletes (Creswell and Poth, 2018). The interviews were conducted face-to-face at times convenient for the athletes, and each interview lasted approximately 40–60 min. During the interviews, a semi-structured interview guide was used to enable participants to express their experiences in depth, and all interviews were audio-recorded. Some example questions asked to the athletes include: “How do you describe your process of starting amputee football and your sports background?”, “How does being able to make your own decisions freely affect your motivation?”, “How competent do you feel in the sports environment, and how has this feeling developed?”, “How do you describe your relationships with your teammates and coaches?”, “How do you cope with the challenges you face during training and competition processes?”, and “What are the most important changes that amputee football has brought to your life?” Open-ended interview questions allowed participants to express their perceptions, experiences, and meaning-making processes (Roulston, 2021). During the interviews, follow-up questions were asked depending on participants’ responses in order to increase data depth. The data collection process continued until meaningful and recurring themes were obtained from the participants. In addition to interview data, non-participant observations were conducted during training sessions and competition processes in order to contextualize athletes’ interpersonal interactions, communication patterns, emotional reactions, and team dynamics. Observational notes were not treated as an independent data source; rather, they were used to support interpretation of interview narratives, strengthen contextual understanding, and enhance analytical depth during theme development.

### Data analysis and trustworthiness

The researcher transcribed the audio recordings obtained from the interviews in order to carry out the data analysis process. To ensure the accuracy and integrity of the data, the transcripts were read multiple times by the researcher, and participant statements were included in the analysis process while preserving their context. Thematic analysis was used in the analysis of the data (Braun and Clarke, 2006). The analytical process followed the six phases proposed by Braun and Clarke (2006): (1) familiarization with the data, (2) generation of initial codes, (3) searching for themes, (4) reviewing themes, (5) defining and naming themes, and (6) producing the report. Accordingly, the data were systematically examined, meaningful units of data were identified, and these units were coded using an open coding approach. In the initial stage of coding, all interview data were organized and coded using MaxQDA 2025 software, which enables qualitative data analysis. Examples of initial codes included “pressure during decision-making,” “fear of making mistakes,” “proving oneself,” “feeling respected,” “restricted autonomy,” and “trust-based belonging.” These codes were subsequently grouped into broader conceptual categories and themes. For example, codes such as “pressure during decision-making,” “feeling controlled,” and “panic under pressure” were brought together under the theme “Performance Pressure and Internal–External Tension.” Similarly, codes including “feeling valued,” “trust,” and “respectful relationships” contributed to the construction of the theme “Social Bonds and Belonging.” The resulting codes were grouped based on similarities and differences, and themes and sub-themes were generated. During this process, the researcher focused particularly on identifying patterns of meaning related to the needs for autonomy, competence, and relatedness.

The researcher also adopted a reflexive position throughout the study. Since the researcher has an academic background in adapted physical activity and disability sport studies, particular attention was paid to minimizing interpretive assumptions during both data collection and analysis processes. Throughout the analysis, the researcher repeatedly returned to the raw data, reviewed codes and themes critically, and reflected on how prior theoretical knowledge and experiences could shape interpretation of participants’ narratives. Reflexive notes were maintained during the coding and theme development stages in order to increase analytical awareness and transparency.

To increase the reliability and credibility of the study, several strategies were employed. Expert opinions were consulted during the development of the interview form to ensure content validity of the questions. Throughout the data collection and analysis process, the researcher carefully conducted the coding and theme development stages and repeatedly reviewed the data to ensure consistency of the findings. In addition, some transcripts belonging to participants were shared with them, and they were asked to confirm the accuracy of the findings (member checking). Credibility was further strengthened through prolonged engagement with the data, triangulation between interview narratives and observational notes, and continuous comparison of codes and themes throughout the analysis process. Dependability was supported by systematically documenting coding decisions, analytical procedures, and theme development processes. Confirmability was enhanced through reflexive memo writing, preservation of raw data and coding records, and consultation with qualitative research experts during theme review stages. The external validity of the study was ensured through data saturation, and the interviews were continued until no new themes emerged.

## Results and discussion

This study examines the experiences of athletes playing amputee football in Türkiye, focusing on processes shaped within the context of autonomy, competence, social relationships, and performance pressure in the sports environment. It provides a perspective on how athletes’ experiences are interpreted through their ability to make their own decisions, feel competent, and establish relationships with their social environment. Based on the data, five themes were identified: (a) autonomy and freedom of decision-making, (b) perception of competence and experiences of success, (c) social bonds and belonging, (d) coping with disability and psychological resilience, and (e) performance pressure and internal–external tension. Although these themes are presented separately for analytical clarity, the findings indicate that athletes’ experiences are highly interconnected and shaped by ongoing tensions between autonomy and control, belonging and selectivity, competence and vulnerability, and motivation and pressure. Therefore, the themes should not be interpreted as isolated categories but as overlapping psychological and social processes constructed within the elite sport environment. These themes are presented below in relation to the literature, together with participants’ narratives.

### Autonomy and freedom of decision-making

This theme covers amputee football players’ experiences regarding their ability to make and implement their own decisions in both daily life and sporting performance processes. Participant narratives indicate that autonomy is not only a preference for athletes but also a strong source of motivation. Athletes reported feeling more motivated, confident, and committed to the process when they were able to make their own decisions. One participant, Mehmet, emphasized the direct effect of autonomy on action by stating, “when I decide something, I do it.” Similarly, another participant noted that having control over his life is an “extra motivation” for him (Aslan). These findings are consistent with the literature on Self-Determination Theory, which suggests that satisfaction of the need for autonomy increases individuals’ intrinsic motivation and well-being (Li et al., 2025). However, the data also reveal that autonomy is not always a stable condition and is often interrupted by external factors. In particular, pressure from coaches, teammates, or the surrounding environment negatively affects athletes’ decision-making processes. One participant explicitly stated, “when I am under pressure, I cannot do that task” (Özgür), while another reported that he “panics” under pressure and that this affects his performance (Can). This demonstrates that the need for autonomy is sustained not merely by its presence but also by supportive environmental conditions.

Similarly, the literature emphasizes that individuals’ needs for autonomy, competence, and relatedness vary depending on the social context and that environmental support plays a decisive role in satisfying these needs (Deci and Ryan, 2000; Van den Broeck et al., 2016; Teixeira et al., 2012). In this sense, although the sports environment is considered a domain that can support autonomy, it may also restrict it through control, surveillance, and performance expectations.

From the perspective of Self-Determination Theory, these findings suggest not only reduced autonomy satisfaction but also experiences of autonomy frustration. According to Bartholomew et al. (2011), need frustration occurs when individuals experience their psychological needs as actively obstructed or controlled by environmental conditions. In the present study, athletes’ descriptions of pressure, imposed decisions, panic, and externally controlled action indicate that autonomy is not merely unsupported at times, but actively constrained by interpersonal and performance-related demands. This demonstrates that elite amputee football environments may simultaneously function as spaces of empowerment and spaces of control.

More strikingly, some athletes defined autonomy as “not listening to anyone” or “doing what I want.” Aslan stated, “I don’t listen to anyone… I do whatever I want,” constructing autonomy as an action space independent of social relationships. This suggests that autonomy may sometimes shift from a healthy self-determination process toward detachment from social bonds or forms of resistance. Therefore, in this study, autonomy emerges not only as a need to be supported but also as an experience that is reconstructed and interpreted differently depending on context.

Yasin expresses that conforming to others’ expectations eliminates autonomy by stating: “What they say… you end up doing what they want; you actually destroy yourself. Not everyone’s words should be listened to. After all, you are living your own life. I think it affects negatively.” Adem uses autonomy as a “boundary-setting” tool depending on the quality of social interaction: “When I feel that the people I regularly interact with do not like me… I draw a line against them. I continue my relationships according to that line.” Musa defines autonomy as a space free from external interference, stating that he is more productive when there is no intervention: “Without anyone interfering, I try to make my own decisions as quickly as possible.” When athletes perceive situations as “orders” or “pressure,” autonomy can turn into active resistance rather than passive compliance. Aslan constructs autonomy as a rejection of authority and a domain of resistance: “I don’t take orders from anyone… I always do what I want, so this is always an extra motivation for me.” Mehmet reports that he only accepts external guidance after filtering it through a “process of questioning,” otherwise he resists: “Because I am a stubborn person, I first ask for the reason. If it makes sense, I do it.” Özgür emphasizes that externally imposed directions undermine autonomy and change the nature of action: “When it is said by someone else, it is different from your own free thought. It feels like an order, so I inevitably feel under pressure.”

Importantly, these findings also reveal a tension between autonomy and relatedness. While athletes seek to protect their autonomy against controlling environments, this sometimes results in distancing themselves from social relationships or limiting interpersonal openness. In this regard, autonomy does not always coexist harmoniously with belonging; rather, athletes continuously negotiate between protecting the self and maintaining social connection. This finding extends existing SDT literature by demonstrating that psychological needs may interact in contradictory ways rather than functioning as uniformly compatible dimensions.

In conclusion, these findings show that autonomy is a strong source of motivation for amputee football players, yet it is continuously challenged by performance pressure and social relationships within the sports environment. Moreover, autonomy is not only a psychological need but also a process through which individuals construct boundaries and protect the self against their social environment. This highlights the importance of autonomy-supportive environments emphasized in the literature, while also indicating a significant gap regarding the ways in which autonomy is sometimes restricted or reshaped in sport contexts.

### Competence perception and experience of success

This section presents the experiences of amputee football players regarding the extent to which they feel competent, skilled, and successful. Participants emphasized that competence is not limited solely to physical performance; rather, it is closely associated with psychological resilience, motivation for development, and social evaluation processes.

A significant proportion of the athletes described feelings of inadequacy not as a reason for withdrawal, but rather as a source of motivation for improvement. Erkan stated that perceived inadequacy does not hinder him; instead, it increases his desire to work harder: “When you know your ability but cannot perform it, I think you should push even more. (…) One should work more, watch more, learn more, and pursue this process more.” Similarly, Eser reported that early in his career he was released from a team because a university considered him insufficient; however, he later re-established his competence through performance, scoring “9 goals in about 8 matches” and later “26 goals, leading his team to the championship.” These experiences indicate that competence is not a stable trait but a continuously reconstructed process shaped by experience and performance outcomes.

Achieved successes emerged as a key factor strengthening athletes’ sense of self-confidence. Can’s Süper Lig championships and national team achievements, Ali’s Champions League results (“Champions League championship”), and Erkan’s international successes (“2–3 European championships”) demonstrate a strong belief in their ability to succeed. These findings are consistent with the literature emphasizing that satisfaction of the need for competence positively influences motivation and well-being (Martela and Ryan, 2018; Zhu et al., 2024). However, the data also reveal that competence perception is a highly fragile construct. Continuous evaluation, comparison, and scrutiny of performance can easily undermine this perception.

This finding can be interpreted within the framework of competence frustration. While SDT literature often focuses on competence satisfaction as a source of confidence and motivation, the present findings show that elite amputee football players are also exposed to constant performance monitoring and comparison processes that threaten their sense of effectiveness. Athletes’ fear of making mistakes, concerns about proving themselves, and anxiety regarding performance visibility indicate that competence is experienced as psychologically unstable and vulnerable to external judgment. In this regard, competence in elite disability sport is not only developed through achievement but also continuously defended against the possibility of inadequacy and exclusion.

In particular, pressure situations or inability to perform effectively lead to decreased confidence and performance decline. Can described this as follows: “I sometimes panic. Especially during competitions, when I feel pressure related to responsibility, I experience panic.” Other participants similarly highlighted that performance is not only a physical process but also a psychological one. Another prominent finding is that some athletes directly associate competence with physical readiness. Especially during the pre-season period or when they do not feel physically prepared, their self-confidence decreases. For instance, Aslan explained his experience during pre-season preparation as follows: “Yes, I feel inadequate, incompetent. (…) I am skilled, but my body is not ready, my mind is not ready.”

This indicates that competence perception depends not only on skill but also on bodily readiness and its continuity. Literature similarly suggests that satisfaction of the need for competence has a direct effect on performance and well-being; however, this need can be easily undermined by social environment, coaching behaviors, and performance expectations.

At the same time, the findings demonstrate a conceptual overlap between competence and performance pressure. Athletes’ self-worth is often linked to visibility, playing time, and external validation, suggesting that competence is socially evaluated rather than internally stabilized. This creates a contradictory process in which athletes seek development and confidence while simultaneously experiencing fear of failure and emotional strain.

In this regard, while sport provides athletes with opportunities for development, it simultaneously creates an environment of continuous evaluation and comparison that may threaten their sense of competence. In conclusion, these findings demonstrate that amputee football players’ competence perceptions have both empowering and fragile dimensions. Athletes feel competent through success experiences and personal development processes, while performance pressure and social evaluation mechanisms may weaken this perception. This highlights an important gap in the literature, where research often emphasizes the positive outcomes of competence need satisfaction but gives limited attention to how this sense of competence becomes vulnerable under contextual pressures.

### Social bonds and belonging

This theme explores how relationships established by amputee football players with their families, friends, teammates, and broader social environment shape both their sporting motivation and their sense of being accepted and valued. Participant narratives indicate that social bonds are not merely a source of emotional support; rather, they provide a foundation of belonging constructed through trust, respect, understanding, and acceptance. However, this sense of belonging is not always stable or inclusive; instead, it is often experienced as selective, fragile, and relationship-dependent.

For some participants, the strongest aspect of social relationships lies in the ability to form trust-based, genuine connections. Ali clearly stated that friendships only become meaningful when trust is present, emphasizing that “I cannot build a bond if I do not trust.” He further explained that being cared for and having his emotions valued by others increases his motivation and makes him feel more positively oriented toward life. This narrative illustrates that the need for relatedness is not limited to mere social contact; rather, it deepens when individuals feel understood, valued, and emotionally acknowledged.

Similarly, for Eser, the defining element of social relationships is not only communication but also being treated with respect. He expressed that he expects mutual respect when he respects others, and that only under such conditions does he feel a sense of achievement. In his account, being accepted does not simply mean being physically present in an environment; it means occupying a role as someone who is valued, listened to, and taken seriously. He further noted that being invited to speak in a well-prepared setting makes him feel good and creates the perception that “people enjoy listening to us.” This suggests that belonging is not only constructed within the team environment but is also symbolically produced through broader social encounters.

However, the data also show that social bonds are not experienced as wide and inclusive networks by all participants. Aslan defines the concept of a “real friend” in a highly limited sense, stating that he has only one person he truly considers a friend and that he does not easily allow new people into his life. This indicates that for some athletes, belonging is constructed not through an expanding social network but through a narrow yet highly intense trust relationship. Importantly, being part of a sports team does not automatically translate into deep belonging; rather, meaningful connection requires selective and deliberate relationship-building.

On the other hand, the positive quality of social relationships is not only related to close ties but also to the emotional impact of everyday interactions. Adem stated that spending time with people he likes and feels happy around positively influences his other social relationships as well. At the same time, he emphasized that acceptance cannot be expected to be uniform across all contexts, highlighting that each person and relationship is different. This underscores that belonging is not a fixed state but a context-dependent and continuously reconstructed experience.

These findings are consistent with Self-Determination Theory, which identifies relatedness as a fundamental psychological need essential for well-being. Interpersonal support has been shown to strengthen autonomy, competence, and particularly relatedness satisfaction, thereby positively influencing motivation, well-being, and performance (Ryan and Deci, 2000; Van den Broeck et al., 2016). Similarly, para-athlete literature highlights that interactions with teammates, social acceptance, and supportive environments can enhance individuals’ sense of social belonging (McLoughlin et al., 2017; Zabala-Dominguez et al., 2023). However, this literature also indicates that in highly competitive sport contexts, para-athletes may face stress, unfair expectations, and psychological pressure, meaning that social support does not emerge automatically or consistently (Dehghansai et al., 2021).

In the present study, belonging is not experienced as unconditional inclusion, but as a selective and relationally negotiated process. Athletes repeatedly emphasize trust, emotional safety, and reciprocity as prerequisites for social connection. This demonstrates that relatedness satisfaction may coexist with experiences of guardedness, relational distance, and selective openness. In this sense, the findings challenge idealized assumptions within sport participation literature that team membership automatically generates belonging and social inclusion. Instead, belonging emerges as fragile, conditional, and dependent on the quality of interpersonal relationships.

At this point, the present study makes an important contribution. The findings demonstrate that belonging among amputee football players is not a uniform or continuously experienced phenomenon; rather, it is a selectively constructed relational process grounded in respect, trust, and emotional reciprocity. In other words, social connection for athletes cannot be reduced to “being part of a team”; it requires being truly seen, having one’s emotions acknowledged, and experiencing relationships built on tested trust. In this sense, the study deepens the para-sport literature by shifting the focus from the mere presence of social support to the question of which relationships actually produce belonging.

Importantly, the findings also reveal a contradiction between collective participation and individual emotional security. Although amputee football is structured as a team sport, athletes do not automatically experience collective belonging. Rather, they carefully evaluate interpersonal trust and selectively determine who can be emotionally close to them. This suggests that social participation alone does not guarantee psychological relatedness, highlighting an important conceptual distinction between physical inclusion and emotionally experienced belonging.

In conclusion, these findings show that social bonds play a central role in athletes’ motivation, happiness, and sense of self-worth; however, these bonds are not always broad, stable, or inclusive. Belonging, in the context of this study, is not an automatic outcome of team membership but a fragile social experience constructed through trust, respect, and being understood. This points to an important gap in the literature, which has often focused on the presence of participation and support, while paying less attention to the quality and selectivity of belonging in amputee football contexts.

### Coping with disability and psychological resilience

This theme focuses on how amputee football players cope with the psychological, social, and emotional difficulties associated with disability and how sport contributes to resilience processes. Participant narratives indicate that sport is not only a physical activity for athletes but also a mechanism through which they reconstruct self-confidence, challenge social stigma, and maintain psychological strength.

Many participants described amputee football as a turning point that transformed their perspective toward disability. Eser stated that before sport he experienced emotional withdrawal and social isolation, whereas participation in football enabled him to become more socially active and confident. Similarly, Mehmet emphasized that sport taught him “not to give up” and strengthened his psychological endurance against difficulties encountered in life. These findings indicate that sport contributes not only to physical adaptation but also to emotional reconstruction and identity development.

Some participants also described psychological resilience as emerging through experiences of proving themselves to others and overcoming social prejudice. Aslan stated that social reactions toward disability initially affected him negatively, but achievements in sport later transformed these perceptions and strengthened his self-confidence. Likewise, Adem explained that being recognized as a successful athlete rather than being defined primarily through disability increased his motivation and psychological strength.

However, this discourse of “self-proving” does not always function as an empowering experience. For some participants, it also generates a persistent pressure to demonstrate competence and defend oneself against societal expectations (Mehmet). In this context, psychological resilience is experienced not only as an internal strength but also as an ongoing process of negotiation and struggle against external norms. This suggests that the success of disabled athletes is often shaped not only by individual effort but also by structural expectations imposed upon them.

From an SDT perspective, these findings indicate that psychological resilience cannot be interpreted solely through positive adaptation or motivation. Rather, resilience develops under conditions where athletes simultaneously experience both need satisfaction and need frustration. While sport may support autonomy, competence, and relatedness, it may also reproduce pressure, evaluation, and expectations that emotionally burden athletes. This demonstrates that resilience in elite amputee football is relationally and structurally produced rather than existing as an isolated individual trait.

Another important finding is that athletes often use social comparison processes in constructing resilience. Some participants explained that observing teammates who experienced more severe physical difficulties increased their appreciation for their own situation and motivated them psychologically. This indicates that resilience is shaped not only by individual coping mechanisms but also by collective experiences and interpersonal observation processes within the team environment.

The findings are consistent with previous literature emphasizing that sport participation among individuals with disabilities positively contributes to psychological resilience, self-esteem, and adaptation processes (Jaarsma et al., 2014; Martin Ginis et al., 2016). However, the present findings also reveal a more complex picture. Psychological resilience among amputee football players is not simply a stable psychological trait emerging naturally from sport participation; rather, it is continuously reconstructed through interactions with performance expectations, social evaluation, and interpersonal support systems.

At this point, a critical distinction emerges. While the literature often conceptualizes psychological resilience as an individual trait, the findings of this study demonstrate that resilience is also shaped by social and structural conditions. In other words, athletes are expected to be resilient; however, the conditions under which this resilience is produced are rarely questioned.

This finding contributes analytically by shifting attention from “successful adaptation” toward the emotional and structural costs of maintaining resilience. Athletes are not only coping with disability itself, but also with performance expectations, social evaluation, and the pressure to continuously demonstrate strength and capability.

In conclusion, the findings demonstrate that amputee football contributes significantly to athletes’ psychological resilience processes by increasing self-confidence, strengthening social participation, and providing opportunities for identity reconstruction. However, resilience is not produced solely through individual psychological strength; it is also shaped by social relationships, recognition, and performance expectations within the sports environment. Therefore, sport simultaneously functions as both a protective and demanding context for disabled athletes’ psychological well-being.

### Performance pressure and internal–external tension

This theme addresses the emotional, cognitive, and interpersonal tensions experienced by amputee football players under conditions of competition, external expectations, evaluation, and pressure. Participant narratives reveal that while sport provides motivation, achievement, and empowerment, it also creates environments characterized by control, stress, fear of failure, and emotional burden.

Many athletes described performance pressure as negatively influencing both decision-making and concentration processes. Özgür stated that when external pressure increases, he becomes unable to carry out tasks effectively, while Can explained that pressure situations create panic and directly reduce his performance. Similarly, Musa reported that excessive intervention from others disrupts his focus and decision-making ability. These findings indicate that athletes’ psychological experiences are shaped not only by internal motivation but also by interpersonal and environmental control mechanisms.

One of the strongest dimensions of performance pressure emerges through coaching behaviors and expectations within the team environment. Some participants stated that instructions given in a highly controlling or commanding style increase emotional tension and create discomfort. Mehmet emphasized that he first questions instructions internally before accepting them, whereas Aslan openly resisted externally imposed authority by stating that he “does not take orders from anyone.” These narratives suggest that athletes are not passive recipients of pressure; rather, they actively negotiate, resist, or reinterpret external expectations.

Such interventions interrupt decision-making processes and create cognitive overload.

Within SDT, these findings can be interpreted as clear examples of need frustration, particularly autonomy frustration and competence frustration (Bartholomew et al., 2011). Athletes describe situations in which their decision-making processes are interrupted, externally controlled, or emotionally pressured by coaches, teammates, and competitive expectations. This demonstrates that performance pressure is not simply a motivational factor associated with elite sport, but an interpersonal and structural condition capable of actively undermining athletes’ psychological needs.

In addition, the fear of making mistakes emerged as a significant source of psychological strain. Athletes frequently expressed concerns about disappointing teammates, losing their position in the team, or failing to meet expectations during competition. These fears increase emotional stress and weaken confidence under pressure situations.

Athletes’ fear of making errors further complicates concentration and negatively affects performance.

Importantly, this theme differs conceptually from the competence theme. While the competence theme focuses on how athletes construct and evaluate their sense of effectiveness and success, the present theme specifically addresses the emotional, cognitive, and interpersonal strain produced by external expectations and controlling environments. In this sense, performance pressure is not merely a consequence of competence evaluation, but a broader structural process shaping athletes’ emotional experiences and motivational regulation.

Another important finding is that athletes simultaneously experience intrinsic motivation and external pressure. While many participants emphasized that they participate in amputee football because they enjoy the sport and feel psychologically strengthened through it, they also acknowledged that competition, expectations, and visibility create emotional tension. This reveals that motivation in elite disability sport environments is not purely autonomous or externally controlled; rather, it exists within a dynamic tension between these dimensions.

The literature similarly emphasizes that controlling environments and external evaluation may undermine athletes’ psychological need satisfaction and negatively affect well-being (Bartholomew et al., 2011; Shannon et al., 2023). At the same time, competitive sport inherently includes evaluation, comparison, and performance expectations. Therefore, the present findings demonstrate that elite amputee football environments simultaneously contain both supportive and psychologically demanding elements.

However, the data show that intrinsic motivation and external pressure are often experienced simultaneously and in tension. While athletes wish to participate in sport based on their own will, they must also adapt to external factors such as coaching expectations, intra-team competition, and performance evaluations. This sometimes leads athletes to suppress their own decisions in favor of external demands.

This contradiction represents one of the most important analytical findings of the study. Athletes simultaneously experience empowerment through sport participation and emotional strain produced by elite performance environments. Therefore, amputee football should not be interpreted solely as a positive rehabilitation or inclusion context, but as a psychologically complex environment in which motivation, pressure, belonging, and control continuously intersect and conflict.

In conclusion, the findings indicate that performance pressure among amputee football players is not only related to competition outcomes but also closely linked to interpersonal relationships, coaching styles, and the emotional demands of elite sport culture. Although sport provides motivation and empowerment opportunities, it simultaneously creates environments where psychological needs may be restricted or frustrated. This highlights the importance of developing athlete-centered and autonomy-supportive approaches within disability sport contexts.

## Conclusion

This study examined the experiences of amputee football players within the framework of Self-Determination Theory, revealing how the psychological needs of autonomy, competence, and relatedness are produced, supported, or constrained within the sport environment. The findings demonstrate that sport is not merely a physical activity context; rather, it represents a multidimensional space in which individuals construct meaning, negotiate their social positioning, and interact with their psychological needs.

The findings show that autonomy functions as a strong source of motivation for athletes, yet this autonomy is continuously negotiated under conditions of performance pressure, interpersonal control, and social expectations. Competence emerges as both empowering and fragile, as athletes constantly reconstruct self-confidence through achievement while simultaneously facing fear of failure and external evaluation. Similarly, relatedness and belonging are not experienced as unconditional inclusion; rather, they are selectively constructed through trust, reciprocity, and emotionally meaningful relationships.

From a theoretical perspective, the study contributes to contemporary Self-Determination Theory literature by demonstrating that psychological needs in elite disability sport contexts cannot be understood solely through linear satisfaction-based models. Instead, autonomy, competence, and relatedness emerge as dynamic, unstable, and context-dependent processes shaped simultaneously by support and frustration. Autonomy may function as resistance against control, competence may become fragile under constant evaluation, and belonging may operate selectively rather than inclusively. These findings extend SDT discussions beyond motivational enhancement by highlighting the contradictory and negotiated nature of psychological need experiences among amputee football players.

The study also demonstrates that amputee football simultaneously functions as a context of empowerment and psychological strain. While sport supports self-confidence, resilience, and social participation, it also reproduces emotional tension, evaluation pressure, and vulnerability. In this regard, athletes’ experiences should not be reduced to simplistic narratives of rehabilitation or inspiration. Rather, elite disability sport environments contain complex interactions between support, pressure, inclusion, and control.

From a practical perspective, the findings highlight the importance of autonomy-supportive coaching approaches, emotionally safe team environments, and psychologically sustainable performance cultures within disability sport contexts. Coaches, sport psychologists, and sport organizations should recognize that athletes’ well-being depends not only on participation opportunities but also on how psychological needs are socially and structurally shaped within the sport environment.

Finally, this study highlights an important gap in the literature on disabled athletes: beyond the existence of participation, there is a need to further examine how participation is experienced, under what conditions it is produced, and for whom it is truly inclusive. In this sense, the study contributes to rethinking inclusivity in sport contexts and provides a critical foundation for future research.

## Data Availability

The raw data supporting the conclusions of this article will be made available by the author, without undue reservation.
